# Encephalic nocardiosis after mild COVID-19: A case report

**DOI:** 10.3389/fneur.2023.1137024

**Published:** 2023-02-22

**Authors:** Nadia Bouhamdani, Dominique Comeau, Christine Bourque, Nancy Saulnier

**Affiliations:** ^1^Vitalité Health Network, Dr. Georges-L.-Dumont University Hospital Center, Research Sector, Moncton, NB, Canada; ^2^Faculty of Medicine and Health Sciences, Université de Sherbrooke, Sherbrooke, QC, Canada; ^3^Centre de Formation Médicale du Nouveau-Brunswick, Université de Moncton, Moncton, NB, Canada

**Keywords:** nocardiosis, *Nocardia farcinica*, coronavirus disease 2019 (COVID-19), post-acute sequelae of COVID-19 (PASC), brain abscess

## Abstract

The COVID-19 pandemic and the associated post-acute sequelae of COVID-19 (PASC) have led to the identification of a complex disease phenotype that is associated with important changes in the immune system. Herein, we describe a unique case of *Nocardia farcinica* cerebral abscess in an individual with sudden immunodeficiency several months after mild COVID-19. Intravenous Bactrim and Imipenem were prescribed for 6 weeks. After this, a 12-month course of Bactrim and Clavulin was prescribed to be taken orally, given the *N. farcinica* infection at the level of the central nervous system. This case report highlights the need for future research into the pathophysiology of COVID-19 and PASC immune dysregulation in convalescent individuals. It also draws attention to the need for timely consideration of opportunistic infections in patients with a history of COVID-19.

## 1. Introduction

Severe acute respiratory syndrome coronavirus-2 (SARS-CoV2) is the causal agent of the coronavirus disease 2019 (COVID-19) global pandemic (1). Alarmingly, long COVID or post-acute sequelae of COVID-19 (PASC) can occur several weeks after infection in a subset of individuals and englobes a multitude of health problems (2, 3). This multisystem disease can arise after severe, mild, or even asymptomatic SARS-CoV2 infection (4, 5) and is characterized by the persistence or onset of new chronic symptoms lasting longer than what is ordinary in most cases of viral infection (6, 7). Indeed, this post-infectious syndrome draws a unique parallel with Ebola and SARS-CoV-1 (8, 9), wherein a long-lasting dysregulation of the immune system is observed long after the infection has cleared (10). Notably, flow cytometry analysis of COVID-19 convalescent individuals, both hospitalized and non-hospitalized, demonstrated that numerous adaptive and innate immune cells were decreased, and activation/exhaustion markers were elevated in T- and B-cell populations (11). Significant lymphopenia (CD4^+^ and CD8^+^ cells) in convalescent individuals were also identified by others (10, 12), and these changes in the peripheral immune system could potentially influence how individuals respond to other infections during this post-COVID-19 timeframe (10), potentially rendering some patients in a state of immunodeficiency.

Individuals may become immunocompromised secondary to underlying malignancies, cancer therapeutics, human immunodeficiency virus/acquired immunodeficiency syndrome (HIV/AIDS), in situations of organ transplant, and after receiving a prolonged corticosteroid regimen (13, 14). Immunocompromised individuals are particularly vulnerable to infections, notably nocardiosis for which the causal agent is *Nocardia species*, a ubiquitous soil-dwelling Gram-positive bacteria. *Nocardia asteroids* are most associated with human disease (15); however, the less common *Nocardia farcinica* is associated with a higher risk of dissemination, drug resistance, and by extension, a higher mortality rate (16–18). The lungs are the primary site of *Nocardia* spp. infection and, when limited to the lung, can be treated with antibiotic treatment. This is however not the case when immunosuppression is prolonged, and secondary sites of infection are established (14); in such cases, bacteremia may later manifest as brain abscesses (19). Mortality rates in the central nervous system involvement range from 40 to 87%, despite therapeutic interventions (20, 21).

Herein, we describe a unique case of *N. farcinica* cerebral abscess in an individual with sudden immunodeficiency several months after mild COVID-19.

## 2. Case description

**Figure 1:**
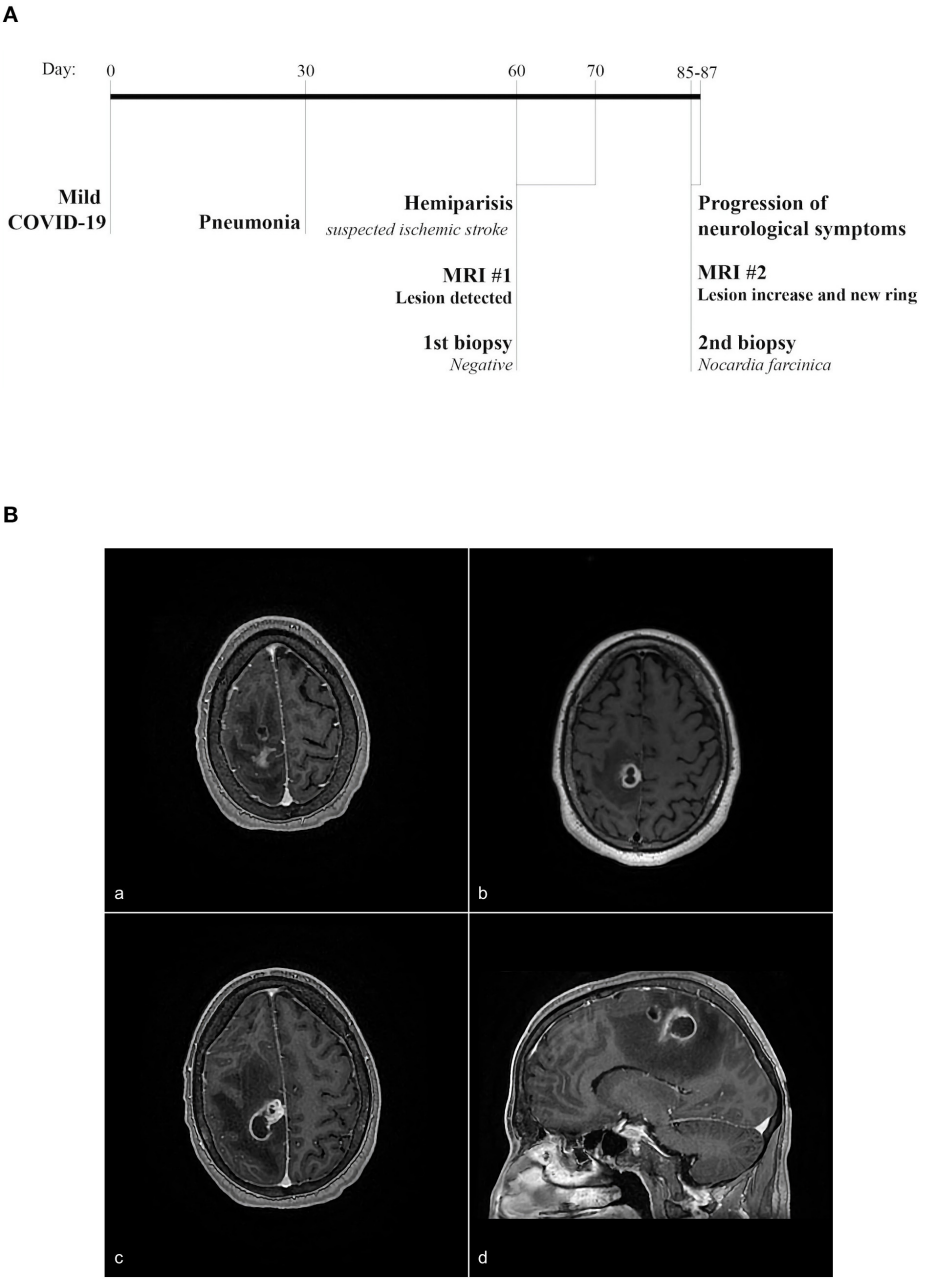
**(A)** Timeline of an episode of care. The patient contracted COVID-19 with mild symptomatology. Thirty days later, the patient was diagnosed with pneumonia and at day 60, presented with hemiparesis. MRI revealed a cerebral lesion. At day 85, the patient presented worsening neurological symptoms and a second MRI showed an additional lesion as well as an increased size of the primary cerebral lesion. At this time, a second biopsy confirmed *N. farcinica*. **(B)** MRI imaging showing an abscess involving the high convexity on the right of the frontal lobe measuring ~1.9 cm × 1.7 cm associated with marked adjacent vasogenic edema **(a, b)**. On repeat imaging, an increase in the size (2.9 cm) of the ring enhancing posterior right frontal/anterior parietal lobe is seen, as well as a new ring-enhancing posterior right frontal lobe lesion measuring 1.1 cm, superior to the lesion described prior **(c, d)**.

**Figure 2:**
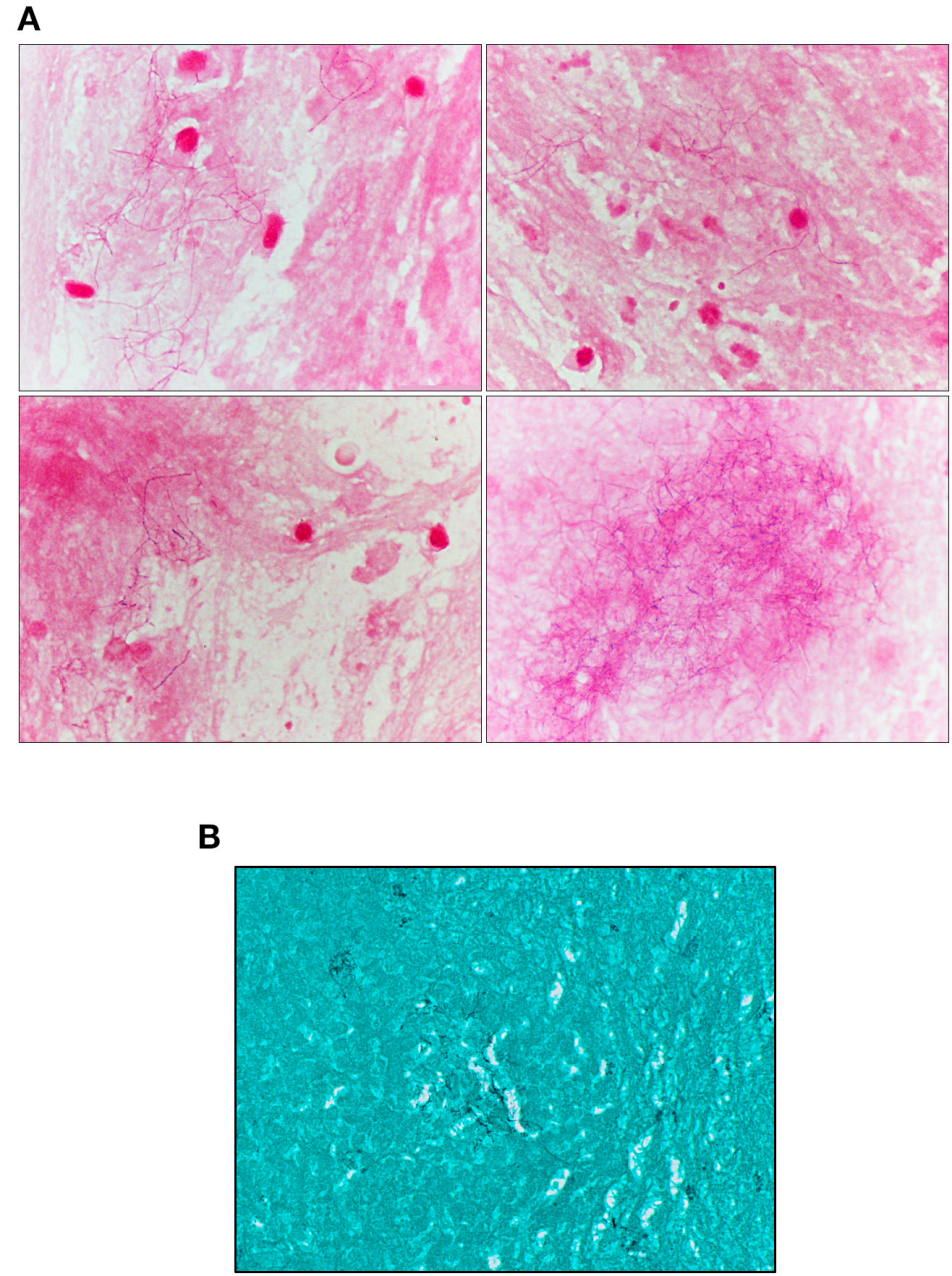
**(A)** Modified acid-fast staining on biopsy showing acid-fast rods. **(B)** Grocott staining.

A male patient in his 50s with a history of high blood pressure, duodenal ulcer, dyslipidemia, and a history of smoking and alcoholism presented at the emergency with left-sided transient hemiparesis. Magnetic resonance imaging showed an enhancing lesion involving the high convexity on the right of the frontal lobe measuring ~1.9 cm × 1.7 cm, associated with marked adjacent vasogenic edema (Figures 1A, B). A biopsy of the lesion highlighted brain parenchyma with reactive gliosis and no significant findings except the growth of *Propionibacterium acnes* in broth cultures. On repeat imaging a couple of weeks later, there was an increase in the size (2.9 cm) of the ring-enhancing posterior right frontal/anterior parietal lobe, as well as a new ring-enhancing posterior right frontal lobe lesion measuring 1.1 cm, superior to the lesion described earlier (Figure 1B). There was also central restricted diffusion. A neurosurgical biopsy showed gliotic brain tissue, necrotic debris, neutrophils, and macrophages within the abscess. Beaded, filamentous bacilli were detected and later confirmed on growth media by Public Health Laboratory to be *N. Farcinica* (Figures 2A, B). Susceptibility results confirmed amoxicillin/clavulanic acid, Imipenem, and trimethoprim–sulfamethoxazole as potentially efficacious against the harvested strain (Supplementary Figure 1).

At 1 month preceding *N. farcinica* cerebral abscess, the patient had contracted pneumonia (inferior right lobe) and was treated with amoxicillin/clavulanic acid, taken orally, twice daily for 10 days. Although the causal agent for the pneumonia was not confirmed to be *N. farcinica*, this route of dissemination (from the lungs to the brain) is very likely as 58% of central nervous system niches originate from the lungs (16). The patient had also contracted COVID-19 1 month before pneumonia, which was confirmed by bedside testing. The patient was fully immunized against COVID-19 (three doses of mRNA vaccine) before contracting the disease.

### 2.1. Investigations

The patient first presented to emergency services for temporary hemiparesis. Given the patient's cardiovascular history, temporary hemiparesis was initially suspected to be the result of an ischemic stroke. After this initial visit, the patient presented three additional times to emergency services over the course of 3 weeks (Figure 1A). Upon the second admission, an MRI was performed, which indicated a cerebral abscess rather than an ischemic stroke (Figures 1A, B). Upon the third admission, a neurological biopsy was negative for bacterial growth, and precautionary antibiotic treatment was prescribed. On the fourth and final visit to emergency services, neurological symptoms had progressed. The growth of the abscess prompted a second neurological biopsy and Public Health Laboratory testing, which revealed the presence of *N. farcinica* (Figure 1B). As cases of *N. farcinica* are mostly found in immunocompromised individuals, immunological flow cytometry analysis of peripheral blood was performed. Indeed, the patient had an immunodeficient profile, but immunosuppression was neither the result of corticosteroid use nor HIV/AIDS. More specifically, lymphopenia was documented in populations CD3^+^CD4^+^, CD3^+^CD8^+^, and CD3^−^CD16^+^CD56^+^, albeit CD19-labeled cells (B lymphocytes) were increased (Table 1). Serum immunoglobulins were all reported to be within the reference range for IgG, IgA, and IgM. Flow cytometry was performed 15 weeks after the patient experienced mild COVID-19 (Table 1).

**Table 1:** Immune assessment.

**Test name**	**Result**	**Ref. range (units)**
CD3 cells/100 cells	66.9	66.6–82.6 (%)
CD3 cells	547^a^	1,047–1,958 (Cell/μl)
CD3^+^CD4^+^ cells/100 cells	53.8	41.4–61.3 (%)
CD3^+^CD4^+^ cells	440^a^	701–1,352 (cell/μl)
CD3^+^CD8^+^ cells/100 cells	12.9^a^	13.4–29.6 (%)
CD3^+^ CD8^+^ cells	105^a^	215–667 (cell/μl)
CD19 cells/100 cells	29.5^a^	6.8–17.0 (%)
CD19 cells	242	105–386 (cell/μl)
CD3^−^CD16^+^ CD56^+^ cells/100 cells	3.1^a^	6.8–19.3 (%)
CD3^−^CD16^+^ CD56^+^ cells	25^a^	133–367 (cell/μl)
CD3^+^CD4^+^ cells/CD3^+^CD8^+^ cells	4.17	
IgA	1.56	0.85–3.85 (g/L)
IgM	1.40	0.53–3.75 (g/L)
IgG	5.90	5.60–17.70 (g/L)

* Abnormal.

### 2.2. Treatment

Intravenous Bactrim and Imipenem were prescribed for 6 weeks. After this, a 12-month course of Bactrim and Clavulin was prescribed to be taken orally, given the *N. farcinica* infection at the level of the central nervous system, especially because of the immunosuppressive state.

### 2.3. Outcomes and follow-up

Antibiotic treatment was effective, and no other issues with infection were experienced afterward. The patient followed a 6-week rehabilitation plan for neurological sequelae and is doing well, despite some residual neuropathy of the left leg.

## 3. Discussion

Corticosteroids are often used in COVID-19-related pneumonia and may lead to an immunocompromised state (22–25) and opportunistic *N. farcinica* infection (26). However, in this case, the patient had not been prescribed any such treatment or other immunomodulators. The immunocompromised state was therefore presumed to have been SARS-CoV2-related. Furthermore, the occurrence of the ailment extended beyond the habitual course of infection and well into PASC territory. The immune response to SARS-CoV2 is believed to be responsible for the enduring symptoms in PASC, potentially through a persisting inflammatory process (27). In this case report, although the immunocompromised state was not typically so severe for opportunistic infection, we believe that the altered immune state in PASC indeed may have enabled *N. farcinica* infection. T-cell lymphopenia was documented, albeit with an accompanying rise in B lymphocytes, as previously documented (10–12). Therefore, we hypothesize that *N. farcinica* infection may potentially have been facilitated by an exhausted immune system; it is becoming increasingly apparent that COVID-19 may lead to an altered immune state and lymphopenia (27–32). Indeed, the immune response to *Nocardia* spp. is mediated by CD8^+^ T cells, whereas B lymphocytes and humoral immunity do not appear to be as important (33), such that the immunocompromised host will be susceptible to such infections (34). Analogously, mucormycosis and links to abnormalities in immune cells after a bout of asymptomatic COVID-19 have also been documented (31, 35). The patient in this study had not received corticosteroid treatments, was not HIV positive, and had similarly contracted COVID-19 but remained mildly symptomatic (31). Hence, it is possible that delayed recovery of T cells may lead to an increased risk of life-threatening infections. Little is currently known about T-cell modulation in mildly symptomatic and asymptomatic disease, as most studies have been carried out in more severe cases of COVID-19.

Furthermore, the considerable systemic inflammation during COVID-19 can lead to endothelitis and disruption of the blood–brain barrier (36, 37), which may have facilitated the entry of *N. farcinica* into the brain. Taken together, both the immunocompromised state and the potential disruption in the blood–brain barrier may have created a propitious environment for the growth and dissemination of the bacterium.

The patient underwent two neurological biopsies to detect bacterial growth. The first biopsy came with the growth of *P. acnes* in the broth only. The second biopsy was sent to a Public Health Laboratory, which identified the causal agent. *Nocardia farcinica* cultures are fastidious, and so, laboratory testing may be negative even in the event of nocardiosis (14); hence, failure to ensure proper growth conditions for an adequate amount of time may fail to reveal growth. Furthermore, if *N. farcinica* had originated from the lungs, the 10-day amoxicillin/clavulanic acid treatment during pneumonia was not sufficient to fully treat the infection as treatment is recommended to last several months (38). A limitation of this case report remains the absence of confirmation of the lungs being the primary site of *N. farcinica* infection. Another important limitation is the lack of understanding regarding the molecular and cellular mechanisms leading to PASC and susceptibility to opportunistic infections. This case emphasizes the importance of early consideration of opportunistic infection in patients with a known history of COVID-19.

This case report highlights the need for future research into the pathophysiology of COVID-19 and PASC immune dysregulation in convalescent individuals. It also draws attention to the need for timely consideration of opportunistic infections for patients with a history of COVID-19.

## 4. Conclusion

This unique case presentation strengthens the notion of immunomodulation after mild COVID-19 and well after the viral infection has cleared. Recognizing these features might prompt considering and testing for infection early on.

## Data availability statement

The original contributions presented in the study are included in the article/Supplementary material, further inquiries can be directed to the corresponding author.

## Ethics statement

The studies involving human participants were reviewed and approved by Vitalité Health Network Research Ethics Board. The patients/participants provided their written informed consent to participate in this study. Written informed consent was obtained from the individual(s) for the publication of any potentially identifiable images or data included in this article.

## Author contributions

NB prepared the ethical submission and paperwork to obtain patient consent. NB and DC wrote and corrected the manuscript. CB and NS coordinated the clinical investigations, patient management, and interpreted the clinical data. All authors reviewed, provided feedback, and approved the final version of the manuscript.

## Supplementary material

Supplementary Figure 1 Antibiogram for *N. farcinica*.
