# Supplementary figures and images for: Encephalic nocardiosis after mild COVID-19: A case report
Source: Front Neurol. 2023 Feb 22;14:1137024. doi: 10.3389/fneur.2023.1137024 (PMC9992866; doi:10.3389/fneur.2023.1137024)

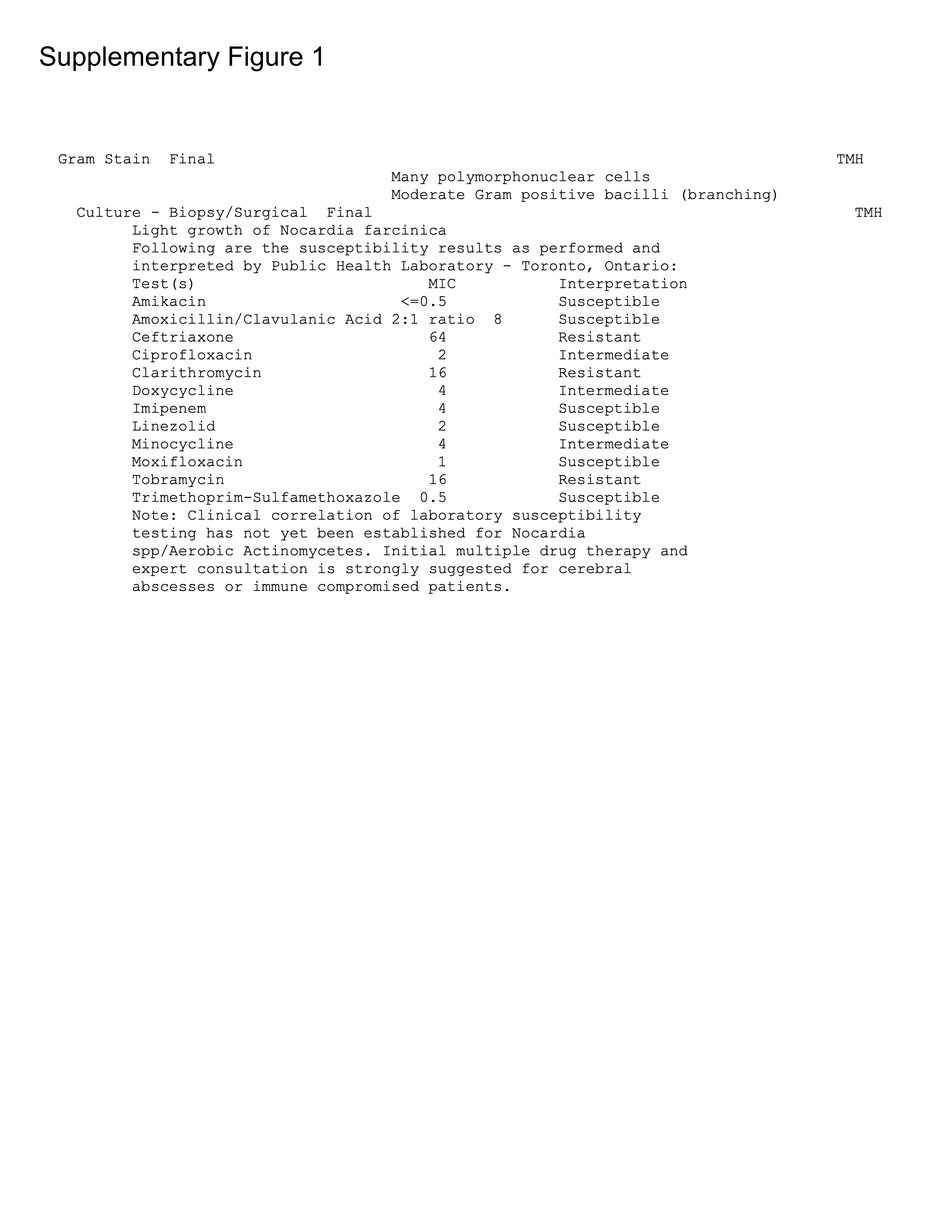

Supplement: Supplementary Figure 1 — Antibiogram for N. farcinica. [file Image_1.TIFF]
